# SOD1 Transcriptional and Posttranscriptional Regulation and Its Potential Implications in ALS

**DOI:** 10.1155/2011/458427

**Published:** 2011-04-17

**Authors:** Pamela Milani, Stella Gagliardi, Emanuela Cova, Cristina Cereda

**Affiliations:** ^1^Laboratory of Experimental Neurobiology, IRCCS, National Neurological Institute “C. Mondino,” Via Mondino 2, 27100 Pavia, Italy; ^2^Department of Neurological Sciences, University of Pavia, 27100 Pavia, Italy

## Abstract

Copper-zinc superoxide dismutase (SOD1) is a detoxifying enzyme localized in the cytosol, nucleus, peroxisomes, and mitochondria. The discovery that mutations in *SOD1* gene cause a subset of familial amyotrophic lateral sclerosis (FALS) has attracted great attention, and studies to date have been mainly focused on discovering mutations in the coding region and investigation at protein level. Considering that changes in SOD1 mRNA levels have been associated with sporadic ALS (SALS), a molecular understanding of the processes involved in the regulation of *SOD1* gene expression could not only unravel novel regulatory pathways that may govern cellular phenotypes and changes in diseases but also might reveal therapeutic targets and treatments. This review seeks to provide an overview of *SOD1* gene structure and of the processes through which SOD1 transcription is controlled. Furthermore, we emphasize the importance to focus future researches on investigating posttranscriptional mechanisms and their relevance to ALS.

## 1. Introduction

Cu/Zn superoxide dismutase or SOD1 is a soluble protein acting as a 32 kDa homodimeric enzyme to convert naturally occurring, but harmful, superoxide radicals to molecular oxygen and hydrogen peroxide. SOD1 is one of the three human superoxide dismutases identified and characterized in mammals: copper-zinc superoxide dismutase (Cu/ZnSOD), manganese superoxide dismutase (MnSOD or SOD2), and extracellular superoxide dismutase (ECSOD or SOD3) [1]. When SOD1 was isolated for the first time, it was thought to be a copper storage protein [2]; the catalytic function of SOD1 was discovered in 1969 by McCord and Fridovich [3], and it was clear that SOD1 acts as a scavenger of superoxide, through a two-step reaction involving reduction and reoxidation of the copper ion in its active site [4]. Primarily, this reaction occurs in the cytoplasm where SOD1 is highly expressed. However, immunohistochemical analysis in rat hepatocytes identified SOD1 in other different subcellular organelles, such as nucleus, lysosomes, and mitochondria [5]. In the 1990s, the scientific community focused their studies on the genetic and biochemical characterization of SOD1 [6], demonstrating that SOD1 plays an important role in diseases as heart failure [7], cancer [8], diabetes [9], Down's syndrome [10], and amyotrophic lateral sclerosis [11]. In fact, in 1984, the first paper about Down's Syndrome was published [12], and in 1993, the first *SOD1* gene mutations associated with ALS were described [11]. In this paper, we focused on *SOD1* gene structure and organization, transcriptional and post-transcriptional gene regulation, and their contributions in the pathogenesis of ALS.

## 2. Gene Structure and Organization

**Figure 1:**
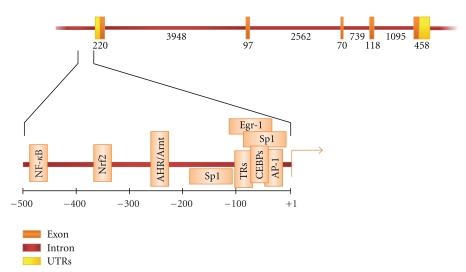
Genomic organization of human *SOD1 *gene. The size of exons and introns, in base pairs, is shown in association with each fragment. The 5′ flanking region is expanded, and the transcription factors, interacting with the corresponding DNA regulatory elements, are shown at the bottom. The transcription start site is depicted as an arrow at position +1.

The human *SOD1* gene (Entrez Gene ID 6647) is located on chromosome 21q22.11, and it codes for the monomeric SOD1 polypeptide (153 amino acids, molecular weight 16 kDa). More precisely, this gene is located from base pair 33,031,935 to base pair 33,041,241 with a genomic size of 9307 bp, according to UCSC Genome Browser (GRCh37/hg19; http://genome.ucsc.edu/). The coding region consists of five exons interrupted by four introns (Figure 1). Several polymorphisms have been identified in *SOD1* gene, mainly distributed in the regulatory regions, including promoter, UTRs, and introns. With regards to SOD1 mRNA splicing, it has been demonstrated that the donor sequence at the first intron presents a T to C variant and, consequently, it deviates from the highly conserved 5′ GT... AG 3′ consensus sites. Nevertheless, it has been demonstrated that this unconventional splice junction (5′ GC... AG 3′) is functional [13]. 

The proximal promoter of human *SOD1 *gene, involved in the basal transcription, has been well studied, and it contains not only the TATA box, but also a CCAAT box and a GC-rich region, which are recognized by CCAAT/enhancer-binding proteins (C/EBPs) and specificity protein 1 (Sp1), respectively [14]. Other binding sites for Egr1, AP1, AHR, Nrf2, NF-*κ*B, and TR transcription factors have been also identified and verified by functional studies (Figure 1). These transcription factors are involved in the regulation of *SOD1* inducible gene expression under specific extra- and intracellular conditions. Different studies performed in rat cellular lines have identified several other regulatory sequences in the rat *SOD1* promoter [15–18]. Considering that the rat *SOD1* gene is very similar to that of human, especially with regard to the proximal part of the promoter region [18], further efforts will be necessary to identify possible other regulatory elements in the human promoter. Moreover, in view of the fact that regulatory elements can be located up to several hundred kilobases away from the gene they control, experiments should be carried out to test whether the long-range control of transcription may represent a mechanism involved in human *SOD1* gene expression. 

With regard to the 5′ untranslated region (5′UTR), Sherman and colleagues [19] demonstrated that SOD1 mRNA posseses various 5′ termini, which are mapped by both primer extension and Sl mapping. The authors showed that the vast majority of the mRNA species has a 5′ start site located 23 bp downstream the TATA box (TATAAA), while the other mRNA molecules have 5′ termini 30, 50, and 65 bp upstream from the major transcription start site. Consequently, these mRNA species are produced through TATA-independent transcription. It would be interesting to perform in-depth investigations about the functional relevance of these multiple transcription start sites, mainly to determine the potential cell and tissue specificity of the different mRNA species. 

**Figure 2:**
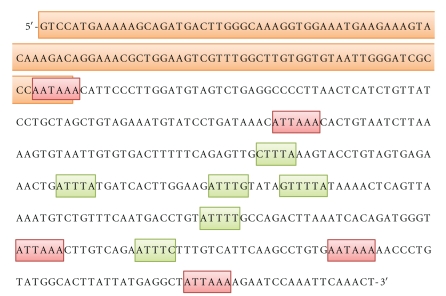
Nucleotide sequence of the fifth exon of *SOD1* gene. The coding portion is highlighted in orange; the polyadenylation sites are in red, and the A/U-rich elements are in green.

Furthermore, two SOD1 mRNAs of about 0.7 kb and 0.9 kb have been identified in a variety of cells, and it has been shown that they are transcribed from the same gene and differ in the length of their 3′UTRs caused by multiple polyadenylation sites. Indeed, analysis of the DNA sequence at the 3′UTR region of the *SOD1* gene revealed the presence of two groups of processing/polyadenylation signals; the first one contains two signals (AATAAA and ATTAAA). The former is fused to the terminal portion of the coding region and it is not utilized. On the contrary, the second site (ATTAAA), localized 76 bp downstream the stop codon, is the one involved in the production of the 0.7 kb mRNA [13] (Figure 2). The second group includes three polyadenylation signals, located 200–250 bp further downstream; the middle one (AATAAA) is involved in the formation of the 0.9 kb SOD1 mRNA (Figure 2). Both the mRNA species are functional, since they can be translated *in vitro* to immunoprecipitable SOD1 proteins [19, 20]. The longer transcript is approximately four times less abundant than the 0.7 kb mRNA.

## 3. Transcriptional Factors Involved in *SOD1* Constitutive and Inducible Expression

Even if *SOD1* has been often considered a “housekeeping gene” due to its high and ubiquitous expression, it is now clear that its induction is fine-tuned modulated by complex intracellular events which probably involve multiple positive and negative regulatory elements acting in concert. Indeed, the diversity of *SOD1* inducers means that there are multiple *cis*-acting elements for this gene, and many studies have been performed to precisely identify the location and the functional relevance of both these DNA sequences and the corresponding *trans*-acting factors. 

### 3.1. C/EBPs (CCAAT/Enhancer Binding Proteins)

These proteins are a family of transcription factors, all containing a highly conserved, basic leucine zipper (bZIP) domain at the C-terminus. It has been demonstrated that C/EBP-related factors are necessary for SOD1 constitutive expression; both C/EBP*α* and C/EBP*β* can interact with the CAAT box (located at position –64 to –55 from transcription start site), playing similar and nonmutually exclusive roles on SOD1 basal transcription [21]. This C/EBP consensus element partially overlaps the Sp1/Egr1 sequence, suggesting that an interlaced network among these transcription factors may act in the fine control of regulation of *SOD1* gene expression.

Furthermore, it has been recently demonstrated that also the transcription factor CCAAT/enhancer binding protein delta (CEBPD, C/EBP*δ*, NF-IL6*β*) is involved in the regulation of human SOD1 transcription [22]. Specifically, CEBPD enhances SOD1 mRNA expression in cisplatin-treated human urothelial carcinoma cell line (NTUB1) via direct promoter transactivation.

### 3.2. Sp1 (Specificity Protein 1)

Sp1 is a ubiquitously expressed C2H2-type zinc finger-containing DNA binding protein. It binds GC-rich motifs (such as 5′-G/T-GGGCGG-G/A-G/A-C/T-3′ or 5′-G/T-G/A-GGCG-G/T-G/A-G/A-C/T-3′) with high affinity and enhances transcription with one of the two glutamine-rich domains [23]. It has been demonstrated that overexpression of Sp1 conspicuously enhances SOD1 basal promoter activity [24].

### 3.3. Egr1 (Early Growth Response-1)

Egr1 is a nuclear phosphoprotein of 80 kDa that functions as a regulator of transcription and belongs to the family of early response genes; it is rapidly induced by mitogens to transduce the proliferative signal. It has also been demonstrated that cytokines and stress signals such as radiation, injury, and oxidative or mechanical stress can induce the expression of this transcription factor [25–29]. In a paper published by Minc and coworkers [30], it has been demonstrated that SOD1 mRNA level is rapidly increased after the treatment of HeLa cells with phorbol-12-myristate-13-acetate (PMA), and the region between nucleotides −59 and −48 has been identified as the one responsible for PMA-induced expression. This region presents noncanonical consensus recognition sequences for Sp1 and Egr-1, and it is bound by Sp1 in a constitutive manner and by Egr1 in response to PMA exposure.

### 3.4. AP1 (Activating Protein 1)

AP-1 is a homo- or heterodimeric transcription factor made by proteins from Jun, Fos, and Maf subfamilies. Activating transcription factor (ATF) proteins also belong to AP1. All these proteins are basic leucine zipper (bZIP) transcription factors. The activity of AP-1 proteins can be regulated by a broad range of environmental cues, including growth factors, cytokines, and oxidative stress, which initiate a variety of intracellular pathways to transduce the information from the extracellular milieu to the nuclear compartment, thus leading to specific cellular responses.

It has been demonstrated that AP1 represses SOD1 transcription by sequestrating essential coactivators, such as Sp1, rather than interacting directly with *SOD1* gene promoter [24].

Furthermore, in agreement with the previous results demonstrating AP1 transcriptional repression activity, it has been shown that neuronal nitric oxide synthase (nNOS) over-expression causes the downregulation of SOD1 in terms of mRNA, protein, and activity levels [31] and that this is caused by two events: the decreased binding of Sp1 to *SOD1* promoter, caused by nNOS interaction with Sp1, and a concomitant increased binding activity of AP1 to the same site.

### 3.5. AHR (Aryl Hydrocarbon Receptor)

AHR is a ligand-activated transcription factor belonging to the helix-loop-helix (bHLH) family. It is well known that, prior to ligand binding, AHR exists in a latent state in the cytosol associated with HSP90 and HSP90 accessory proteins [32–34]. The interaction with HSP90 is fundamental to retain AHR in the cytoplasm. Many synthetic halogenated and nonhalogenated aromatic hydrocarbons activate AHR signal pathway through a direct interaction: upon ligand binding, HSP90-bound AHR translocates into the nucleus where it exchanges HSP90 for another bHLH protein, known as hydrocarbon receptor nuclear translocator (Arnt). This new heterodimeric complex binds to the xenobiotic responsive element (XRE) that functions as a *cis*-acting enhancer in the promoter region of numerous phase I and II drug-metabolizing enzyme genes [35, 36]. Cho and coworkers [37] observed an increased expression of SOD1 mRNA and protein after the exposure of human HepG2 and HeLa cells to one of the most toxic man-made hazard, the 2,3,7,8-tetrachlorodibenzo-p-dioxin (TCDD), an environmental contaminant belonging to the halogenated aromatic hydrocarbons class and interacting with AHR. The authors identified the presence of a xenobiotic responsive element in the 5^'^flanking region of human *SOD1* gene (located between −255 and −238 from the transcription start site), which is responsible for the induction by TCDD.

### 3.6. Nrf2 (Nuclear Factor E2-Related Factor 2)

Nrf2 is a Cap′n′collar (Cnc) transcription factor that regulates the expression and the coordinated induction of a battery of defensive genes encoding phase II detoxifying enzymes and antioxidant proteins. The activity of Nrf2 is controlled by the cysteine-rich cytosolic INrf2 (Inhibitor of Nrf2), also known as Keap1 (Kelch-like ECH-associated protein1). The activation of Nrf2 pathway requires its cytosolic stabilization via oxidative modification of distinct Keap1 cysteine residues, Keap1 preoteasomal degradation, and/or phosphorylation of Nrf2. The dissociation from Keap1 is a prerequisite for Nrf2 translocation to the nucleus. In this compartment, Nrf2 heterodimerizes with a small Maf protein and binds to the *cis*-acting antioxidant/electrophile responsive element (ARE/EpRE), activating the transcription of various cytoprotective genes involved in detoxification from xenobiotics, electrophile conjugation, ROS scavenging, and regulation of intracellular redox homeostasis [38–41].

An antioxidant responsive element (located between −356 and −330 from the transcription start site) has been identified in human *SOD1* gene promoter [42, 43]. Park and colleagues demonstrated that *SOD1 *gene transcription is induced in human HepG2 hepatoma cells after the treatment with the dioxin TCDD, which produces reactive oxygen species thus leading to the activation of Nrf2 signalling. Moreover, it has been shown that low-dose and nontoxic proteasome inhibition enhances mRNA and protein expression of SOD1 in different human endothelial and vascular smooth muscle cells through transcriptional induction mediated by Nrf2 [43].

### 3.7. NF-*κ*B (Nuclear Factor-KappaB)

The term NF-*κ*B refers to a family of five structurally related transcription factors (p50, p52, RelA/p65, c-Rel, and RelB), all containing the Rel homology domain (RHD) within the N-terminus and acting as homo- and heterodimeric DNA binding complexes [44]. Their functionality and nuclear localization are controlled by a family of inhibitor proteins, known as IkappaBs (I*κ*Bs). In nonstimulated cells, NF-*κ*B dimers are bound to inhibitory I*κ*B proteins and are thereby sequestered in the cytoplasm as inactive complexes. Several studies showed that NF-*κ*B activity is induced in most cell types in response to a broad variety of stimuli, ranging from cytokines, radiation, and oxidative stress (such as exposure to H_2_O_2_), with major roles in coordinating innate and adaptive immunity, cell activation and proliferation, survival, development, and apoptosis [45, 46]. NF-*κ*B was one of the first transcription factors shown to be redox regulated [47–50], and Rojo and colleagues [51] showed that cell treatment with H_2_O_2 _initiates the PI3K/Akt cascades, which participates in NF-*κ*B activation and in subsequent SOD1 transcriptional induction. Indeed, the authors identified a p65-NF-*κ*B binding site in the human *SOD1* promoter (GGTAAGTCCC), and they demonstrated that Akt-activated NF-*κ*B presents increased binding to this sequence, mediating the upregulation of SOD1 expression.

### 3.8. TRs (Thyroid Hormone Receptors)

The thyroid hormone receptors are encoded by the TRalpha and TRbeta genes and are ligand-dependent transcription factors, since they bind both thyroid hormones (THs) and TH-response elements (TREs) that are located in the promoters of target genes. These proteins, which belong to the nuclear receptor superfamily, regulate development and a broad variety of critical cellular functions including growth, differentiation, basal metabolic rate, and metabolism of protein, fat, and carbohydrate. It has been demonstrated that TRs can either enhance or repress transcription. Hormone-dependent repression requires binding to negative TREs (nTREs); in particular, the unoccupied receptor increases transcription on nTREs, and ligand binding to TR reverses this induction [52]. The responsive sites of these negatively regulated genes are generally localized to the proximal promoter region [53]. In agreement with this observation, Santos and co-workers [54] have identified a thyroid hormone inhibitory element between −157 and +17 of the human *SOD1 *promoter and demonstrated that T_3_ exposure reverses the induction of SOD1 transcription caused by the ROS-producing paraquat and PMA agents, through the direct TR/T_3_-DNA interaction. On the contrary, *SOD1* promoter is significantly upregulated by unliganded TRs.

## 4. Posttranscriptional Gene Regulation

Historically, efforts aimed at decoding the molecular mechanism of gene expression have been principally focused on transcriptional control. However, post-transcriptional regulation of mRNAs is now considered as an important step in the flow of genetic information providing additional opportunities by which gene expression could be rapidly modulated. Indeed, post-transcriptional events such as mRNA processing and nuclear export, mRNA stability, translational efficiency, and microRNA-dependent modulation create a complex intracellular network contributing to determine the global levels of specific mRNAs. Most mRNA regulatory elements are located within the 5′ and 3′UTRs, where they function as platforms for the binding of numerous proteins and noncoding RNAs. The 5′UTR is principally involved in controlling mRNA translation [55], while the 3′UTR regulates multiple steps of mRNA metabolism and stability. 

Very few works on post-transcriptional regulation of SOD1 have been published so far. 

As mentioned above, two species of SOD1 mRNA with different 3′UTR lengths have been identified, and they produce *in vitro* different quantities of SOD1 protein. Kilk and colleagues [56] performed functional study to investigate the effect of this regulatory region on SOD1 protein expression, and they observed that cells transfected with a long cDNA, containing the long 3′UTR, produce three times more SOD1 protein than cells transfected with a cDNA presenting a deletion of the last 185 bp from the 3′UTR. The authors hypothesized that the ability of the long mRNA to produce more SOD1 enzyme may depend on specific sequences located in the 3′UTR, and they identified the presence of various A/U-rich elements (AREs) in this region (AUUUA, CUUUA, AUUUG, GUUUUA, AUUUU, and AUUUC) (Figure 2). AREs were initially defined by the sequence AUUUA [57], although subsequent studies showed other sequences with U-stretches presenting the same properties as the canonical element. Several ARE-binding proteins, which function as *trans*-acting factors, have been identified, and interaction of these proteins can correlate negatively or positively with the stability and the translatability of the target mRNA [58]. Considering the biological relevance of these sequences, the identification of the proteins potentially interacting with SOD1 mRNA may shed new light on SOD1 gene expression modulation. Moreover, another important question is whether these SOD1 mRNA species, differing for their 3′UTR lengths, could be somehow linked to pathological conditions, since it is possible that specific cellular stresses may vary the relative proportions of such variants with relevant implications for cell phenotypes. 

In addition, also SOD1 post-transcriptional regulation mediated by microRNAs (miRNAs) represents a field still almost unexplored. miRNAs, which are small non-protein-coding RNAs, function as key post-transcriptional regulators of gene expression by usually base pairing to the 3′UTR of the target mRNAs to cause translational repression and mRNA decay [59, 60]. Recently, Wang and co-workers [61] showed, through computational and biological approaches, that SOD1 is a target of miR-377 in human and mouse mesangial cells and miR-377 diminished SOD1 protein levels. Owing to the importance of miRNAs in mRNA metabolism control, future in-depth researches should be carried out in this field to unravel previously unrecognized complex regulatory and interactive pathways that may cooperate to modulate SOD1 quantities and whose dysregulation may be relevant for ALS disease states.

## 5. Amyotrophic Lateral Sclerosis

Amyotrophic lateral sclerosis disease (ALS) is a multifactor and multigenic disorder with still unknown aetiology and pathogenesis. Even if several new genes associated to ALS have been described, *SOD1* gene is considered the major gene involved in ALS pathogenesis. 

### 5.1. Genetic Variants

In 1991, Siddique and collaborators [62] identified a linkage of familial ALS to the *SOD1* locus on chromosome 21q22 and demonstrated genetic locus heterogeneity in FALS studying 23 ALS families. In 1993, Rosen [11] and collaborators have reported tight genetic linkage between ALS and *SOD1* gene, establishing *SOD1* as the first causative gene for ALS (genetic nomenclature, ALS1). Mutations in *SOD1* gene are responsible for 12–23% of all FALS cases [63, 64]. More than 150 *SOD1* mutations have been reported in 68 of the 153 codons, spread over all five exons (ALS Online Genetic Database, ALSOD: http://alsod.iop.kcl.ac.uk/) [65], most of them cause disease. Although most mutations are missense, nonsense mutations and deletions have also been found [66, 67].* SOD1* exonic mutations have occasionally been described in patients with apparently sporadic onset, and it has been estimated that 1% of SALS could be due to *SOD1* mutations [68]. Many of the mutations are at present unique to individual families. Correlation between mutations and phenotype has been investigated, because of a large variability in phenotype in term of disease progression, extramotor features, and age of onset but is generally difficult to predict on the basis of the *SOD1* mutations [66].

### 5.2. SOD1 Gene Expression in ALS

Even if *SOD1* gene has been considered fundamental in ALS, numerous studies have been done concerning protein expression and gene mutations, but mRNA level studies are very rare. For the first time, in 1997, Nishiyama studied SOD1 expression in cervical and spinal cord of ALS patients using a quantitative in situ hybridization technique [69]. There were no significant differences between the amounts of SOD1 mRNA level observed in patients with sporadic or familial disease, and normal control subjects. Moreover, motoneurons in the normal spinal ventral horn and precentral motor cortex exhibited significantly higher levels of SOD1 messenger RNA than did other neurons [69]. In recent years, expression studies often produced conflicting data about up- or down- regulation of ALS relevant genes, such as *SOD1* [70–72]. The confounding data were probably due to differences in the central nervous system (CNS) tissue areas analyzed and possibly differences in the analytical techniques used.

The latest *SOD1* gene expression study demonstrated that SOD1 mRNA level is elevated in specific nervous areas typically affected by ALS disease (i.e., brain stem and spinal cord) and not in other brain areas not involved in the neurodegenerative process (i.e., cerebellum and cerebral cortex) in SALS patients [73]. Moreover, increased SOD1 mRNA expression has been detected in peripheral system as lymphocytes from SALS patients compared to healthy people [73]. However, in the same work, Western Blotting analysis showed lower or similar expression of the protein both in lymphocytes and in nervous tissue-affected areas thus confirming a previous observation obtained in the peripheral cells [74]. On the contrary, histopathological analysis of spinal cord tissue from SALS patients evidenced an increased SOD1 protein expression compared with controls. This result correlates with the evidence of higher mRNA level and suggests the hypothesis that, in at least a subgroup of SALS patients, misfolded and aggregated SOD1 protein would precipitate in the insoluble fraction becoming undetectable after extraction with routinely used lysis buffers. Actually, proteinaceous inclusion bodies that may contain SOD1 have been already described in motor neurons of SALS patients [75–77], and the presence of aberrant SOD1 species associated with human sporadic ALS has been extensively demonstrated [78–80]. Insoluble SOD1-containing aggregates are also a characteristic of the familial ALS linked to SOD1 mutation. In mutSOD1 transgenic mouse, the detergent-insoluble accumulation of SOD1 appears before or coincident with symptom onset [81], and in the human SOD1-associated ALS, mutant SOD1 aggregation is a pathological hallmark [82]. This feature common to familial and sporadic cases suggests a shared pathological trait.

## 6. Conclusions

Considering the few literature data about *SOD1* gene regulation, it appears clear the importance of understanding how the expression of this gene can be controlled and modulated in normal and ALS pathological conditions. The molecular pathways regulating SOD1 expression at the transcriptional level have been studied, and many *cis*-elements and the relative *trans*-acting protein factors have been identified. Nevertheless, the details of most of these interactions and the *in vivo* consequences of their modulation for the most part are yet to be determined, mainly in relation to pathological states. Moreover, also post-transcriptional mechanisms may exert important functions in determining the global levels of functional SOD1. Indeed, the formation of specific ribonucleoprotein complexes and RNA-silencing events may provide additional mechanisms by which SOD1 expression could be rapidly and precisely modulated. In particular, considering that microRNAs are emerging as master regulators of gene expression due to their capability to finely tune gene dosage, investigation in this field should become a preferred topic for new researches. Future gain of knowledge about these processes may help to discover previously unanticipated integrated networks, leading to new and exciting directions in the field of ALS medical research with promising prospects. Indeed, the progress in understanding the mechanisms of transcriptional and post-transcriptional control could offer hope for the development of new-generation drugs or medical treatment strategies.
